# Prime, shock and kill: BCL-2 inhibition for HIV cure

**DOI:** 10.3389/fimmu.2022.1033609

**Published:** 2022-10-20

**Authors:** Aswath P. Chandrasekar, Andrew D. Badley

**Affiliations:** ^1^ Department of Laboratory Medicine and Pathology, Mayo Clinic, Rochester, MN, United States; ^2^ Division of Infectious Diseases, Mayo Clinic, Rochester, MN, United States; ^3^ Department of Molecular Medicine, Mayo Clinic, Rochester, MN, United States

**Keywords:** Bcl-2, hiv, HIV cure, shock and kill strategies, apoptosis

## Abstract

While modern HIV therapy can effectively suppress viral replication, the persistence of the latent reservoir posits the greatest hurdle to complete cure. The “shock and kill” strategy is under investigation for HIV therapy, aiming to reactivate latent HIV, and subsequently eliminate it through anti-retroviral therapy and host immune function. However, thus far, studies have yielded suboptimal results, stemming from a combination of ineffective latency reversal and poor immune clearance. Concomitantly, studies have now revealed the importance of the BCL-2 anti-apoptotic protein as a critical mediator of infected cell survival, reservoir maintenance and immune evasion in HIV. Furthermore, BCL-2 inhibitors are now recognized for their anti-HIV effects in pre-clinical studies. This minireview aims to examine the intersection of BCL-2 inhibition and current shock and kill efforts, hoping to inform future studies which may ultimately yield a cure for HIV.

## Introduction

Modern combination anti-retroviral therapy (cART) is highly effective at suppressing HIV viremia to levels below the limit of detection. However, infected individuals continue to harbor integrated, transcriptionally silent proviral forms in a pool of infected cells known as the latent reservoir (1). Studies have now highlighted the dynamic nature of the reservoir, which can propagate even under the coverage of cART through low level transcription and clonal expansion (2–4). Strategies to target the latent reservoir aim to achieve either complete elimination of infected cells (a sterilizing cure), or permanently prevent HIV transcription in these cells (a functional cure) (5).

The “Shock and Kill” HIV cure strategy centers around the former principle, aiming to reactivate latent HIV, and subsequently eliminate it through anti-retroviral therapy and host immune function (6). However, even in the setting of efficient viral reactivation, numerous barriers have been identified which prevent host immunity from maximally clearing HIV infected cells *in-vivo* including poor immune recognition, immune exhaustion, the effects of immune-modulatory cytokines, and upregulation of prosurvival proteins (7–12).

Through a combination of mechanisms, these changes allow for either infected cell survival, or compromised immune effector function, or both. One such factor that has been recognized to facilitate HIV persistence by both priming infected cells for survival, and simultaneously antagonizing the host immune response, is the BCL-2 pro-survival protein (13). This minireview aims to examine the implications of this protein to HIV pathogenesis and to therapeutic targeting for HIV “shock and kill” efforts.

## The role of BCL-2 in HIV persistence

BCL-2 and its homologs are intracellular regulators of multiple cellular processes, the most crucial of which is cell survival and apoptosis. Prototypical members of the BCL-2 family are characterized by the presence of four conserved homology domains: BH1, BH2, BH3 and BH4 (14). At homeostasis, cell apoptosis is controlled by the balance between BCL-2 (and other anti-apoptotic homologs) and the pro-apoptotic homologs, with a subset of proteins containing only the BH3 domain, serving as modulators of this balance. The proposed mechanisms through which these interactions occur have been reviewed extensively elsewhere (15). The BCL-2 protein prevents cell death through the stabilization of the mitochondrial membrane *via* the sequestration of pro-apoptotic proteins which, canonically, occupy a hydrophobic groove in the BH3 domain of anti-apoptotic BCL-2 family members. The HIV life cycle is dependent on host cell apoptosis to facilitate the release of viral progeny, and various HIV proteins have been shown to modulate the levels of BCL-2 family members to either promote or prevent the death of infected cells. [Reviewed in detail in (13)]

Apoptosis is crucial to the HIV life cycle, and one of the mechanisms through which HIV achieves infected cell apoptosis is through the Casp8p41 pathway. Casp8p41 is a 41 kilodalton, BH3-like protein that is generated due to the cleavage of host procaspase8 by HIV protease (16). Through the direct potentiation of the pro-apoptotic BCL-2 family members BAK and BAX and subsequent mitochondrial membrane depolarization, Casp8p41 results in host cell apoptosis (17, 18). Clinically, it was observed that intracellular Casp8p41 levels in HIV infected individuals correlated inversely with CD4 T-cell counts (19), and that patients on suppressive ART who demonstrated continuous production of Casp8p41 exhibited an increased risk of CD4 losses (20).

Of particular importance was the recognition that latently infected cells produced Casp8p41 upon reactivation, which was observed to be bound by host BCL-2 and degraded by the proteasome, antagonizing its pro-apoptotic phenotype, thereby facilitating HIV survival (21, 22). The ability of BCL-2 to abrogate Casp8p41 induced apoptosis led to the hypothesis that BCL-2 contributed to the establishment of the latent reservoir.

Ex vivo studies have now verified that the cell subsets that are classically described to harbor the latent HIV reservoir, namely central memory T-cells, have been observed to express higher levels of BCL-2 (21), and that BCL-2^high^ cells have been seen to occur more frequently in HIV infected individuals compared to uninfected controls (9). Studies of simian immuno deficiency (SIV) infection in ART suppressed macaques revealed that CTLA^+^ PD1^-^ Memory CD4 cells that harbored significant quantities of replication competent provirus demonstrated significantly higher BCL-2 expression compared to other subsets (23). Most importantly, it has now been recognized that the inducible HIV reservoir is preferentially enriched in cells with higher expression of BCL-2 *in-vivo* (11).

HIV latency has been recapitulated through lentiviral transfection of primary CD4 cells with a BCL-2 construct, in an *in-vitro* model which allowed for sustained HIV replication, and recapitulated changes seen during the natural course of HIV infection, further illustrating the critical role of BCL-2 in HIV latency (24–26).

**Figure 1:**
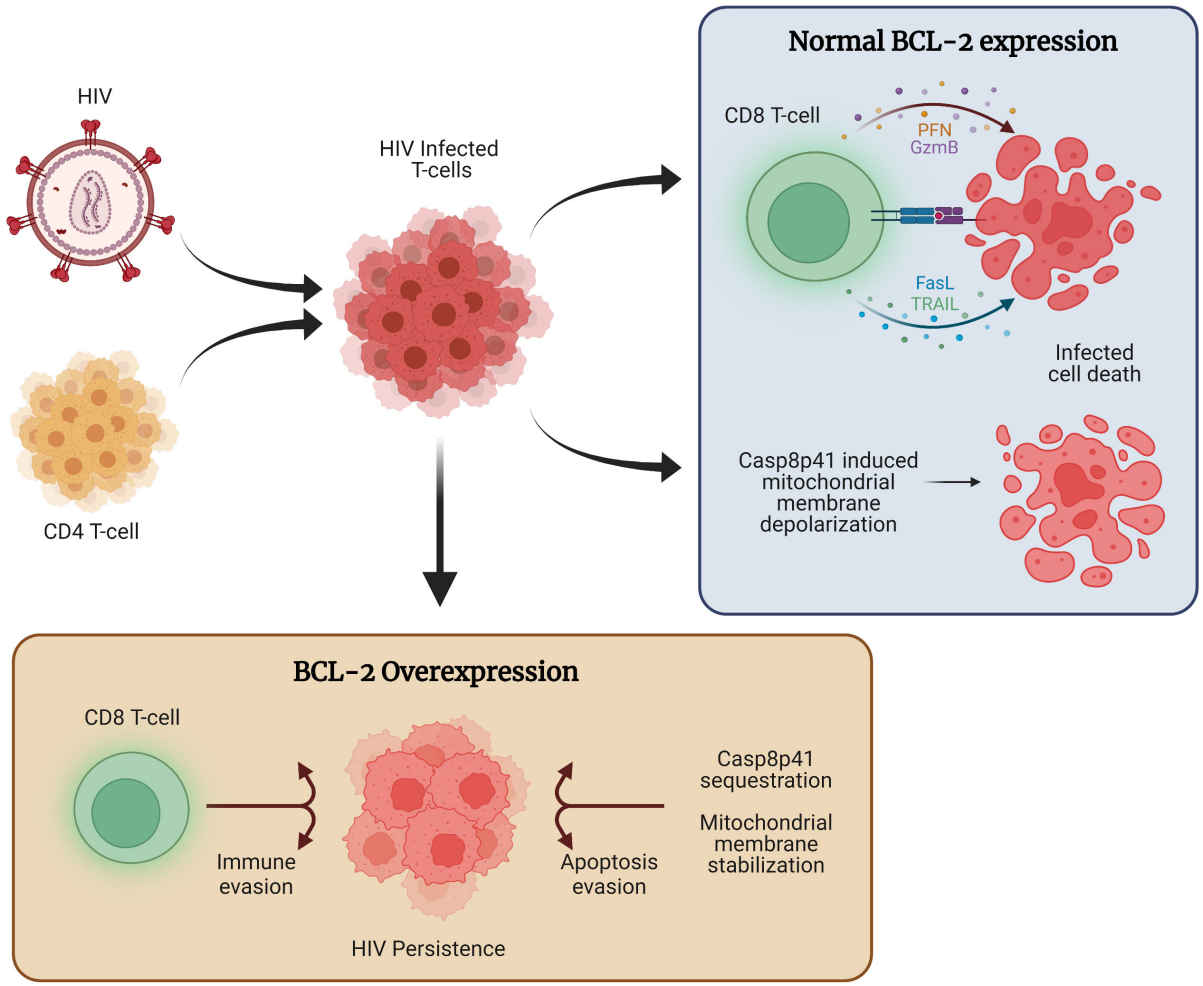
The role of BCL-2 in HIV persistence. Following HIV infection, a combination of host immune function and direct viral cytopathic effects, such as those mediated through Casp8p41 cause the majority of cells to die. However, a subset of infected cells expresses higher levels of BCL-2, allowing for apoptosis evasion and compromised immune function, ultimately allowing for the persistence of HIV infected cells.

Overall, the critical role of BCL-2 for HIV persistence and latency is supported by a growing body of evidence (**Figure 1**).

## The role of BCL-2 in immune cell function

*In-vivo*, cell mediated, cytotoxic immune responses are mediated by cytotoxic CD8 T-cells and Natural killer (NK) cells. These cells achieve target cell apoptosis through either granule mediated, or cytotoxic ligand mediated signalling. BCL-2 serves as a key regulatory factor for both mechanisms (27–30). CD8 and NK cells are crucial HIV control and dysregulated immune response is a key factor leading to the persistence of the HIV reservoir (1). CD8 and NK cells respond to antigenic challenge through overlapping mechanisms which ultimately result in target cell elimination. These mechanisms include granule mediated cytotoxicity and pro-apoptotic ligands such as FasL and TNF-related apoptosis inducting ligand (TRAIL) (27, 31, 32). The intersection of the aforementioned cytotoxic mechanisms utilized by effector cells, and the anti-apoptotic BCL-2 protein, will be examined further below.

Perforin/Granzyme B is a key effector pathway that is utilized by both CD8 T-cells and NK cells (27, 33, 34). Briefly, following the recognition of a cognate antigen, effector cells degranulate, leading to the release of Perforin and Granzyme B. Perforin binds to the target cell wall and causes pore formation in a manner that is postulated to be similar to bacterial cytolysins, allowing for the intracellular delivery of granzyme B (34). Granzyme B initiates the apoptotic singling cascade either through direct activation of caspases, or through the activation of BID. Activated BID subsequently activated the pro-apoptotic BAX protein, which in turn leads to mitochondrial membrane depolarization and efflux of apoptotic mediators, ultimately leading to cell death.

*In vitro* BCL-2 has been seen to antagonize granzyme mediated cell death through the inhibition of downstream Bax activation (35). Additionally, it was observed that BCL-2 strongly antagonized Perforin mediated Granzyme translocation to the nucleus, leading to blunted cytotoxicity (36). Overexpression of BCL-2 in mouse cell lines was seen to inhibit granzyme mediated apoptosis *in-vitro*; however it was observed that cytotoxic T-cells retained their apoptotic functionality, likely through granzyme B independent mechanisms (28). Overall, it has now been well established that BCL-2 overexpression antagonizes apoptosis induced by perforin and granzyme B.

Fas ligand (FasL) is a key effector molecule that is produced by both CD8 and NK cells and has been widely studied in the context of HIV infection and CD4 depletion (27, 33, 37–39). FasL binds its cognate receptor, *Fas*, on the surface of target cells, and recruits the “Fas associated death domain” (FADD) (40) to activate the apoptotic pathway. Fas has been shown to elicit apoptosis through Bid/Bax/Bak dependent and independent pathways in lymphocytes (41). Fas signaling is also a crucial to chimeric antigen receptor T- cell (CAR-T) mediated clearance in acute lymphoblastic leukemia (ALL) (42, 43).

The role of BCL-2 in preventing Fas mediated cell death is controversial and multiple reports have suggested that FasL can act in a BCL-2 independent fashion to induce cell death (44). However, it has also been demonstrated that BCL-2 overexpression can prevent Fas mediated cell death in lymphoid cell lines and in breast cancer (29, 45), and can abrogate cytotoxic T-lymphocyte (CTL) responses in cells overexpressing Fas receptor (46) suggesting that even though Fas signalling can function independently of pro-apoptotic BCL-2 family members, BCL-2 may play a role in blunting the apoptotic effect of Fas (47). BCL-2 has also been shown to downregulate the expression of FasL, through the disruption of the nuclear translocation of nuclear factor of activated T-cells (NFAT), *via* the inhibition of calcineurin, conferring drug resistance to FasL dependent drugs (48).

TNF-related apoptosis inducting ligand (TRAIL) is another key effector molecule that is essential for CD8 and NK function (27, 32). Following the binding of TRAIL to its cognate receptor, TRAIL Receptors 1 and 2, FADD is recruited to initiate apoptotic signalling (49). Similar to Fas, TRAIL too has been identified as a crucial regulator of CAR-T function in lymphoid malignancy (42, 43). TRAIL mediated apoptosis has been described to be directly inhibited by BCL-2 overexpression in multiple cell lines, though similar to FasL, lymphoid cells exhibited apoptosis through a BCL-2 independent pathway (30).

In terms of cell mediated cytotoxicity, upregulation of the BCL-2 homologs MCL-2 and BCL-2A1 were recently described to inhibit cytotoxic T-cell mediated clearance of melanoma cells (50). BCL-2 upregulation in CD8 effector cells in lymphoid malignancies has been demonstrated to directly impair the ability of immune effectors to degranulate efficiently, blunting the immune response. These cells were seen to also co-express PD-1 and TIM-3, both of which have been implicated in HIV immune escape (51–53). It has now also been demonstrated that cytotoxic T cells exhibit impaired clearance of target cells overexpressing BCL-2 (11).

Therefore, given the ability of BCL-2 to antagonize both direct viral cytopathic effects and cytotoxicity mediated by immune effector mechanisms, targeted therapy against BCL-2 has become a topic of growing importance in HIV shock and kill studies. The intersection of these will be examined further below.

## The “shock”: BCL-2 inhibitors and HIV latency reversal

The first pillar of the “shock and kill” strategy is the reactivation of the latent reservoir. Various classes of drugs, known as latency reversal agents (LRAs) are under investigation for the reversal of HIV latency *in-vivo*. These include Histone deacetylase (HDAC) inhibitors, Protein kinase C agonists, second mitochondrial-derived activator of caspases (SMAC) mimetics, Proteasome inhibitors, histone methyltransferase (HMT) inhibitors, DNA methyltransferase inhibitors, bromodomain inhibitors, Toll-like receptor (TLR) agonists and cytokines such as IL-15 super-agonist (N-803) (54, 55).

The crucial role that BCL-2 plays in the persistence of malignancy lead to the development of now clinically approved BCL-2 inhibitors. Given our growing understanding that BCL-2 is also a critical determinant of the HIV latent reservoir, studies have aimed to assess the impact of BCL-2 inhibition on HIV dynamics. Thus far, it has been established that selective BCL-2 inhibition can decrease the number of virally infected cells, decrease the size of the inducible latent reservoir, and restore the ability of HIV specific cytotoxic lymphocytes to target latently infected cells following reactivation (11, 21, 22, 56).

However, a suitable combination of an LRA and BCL-2 inhibitor that allows for maximal reactivation and selective elimination of HIV is yet to be identified. The following text aims to examine the effects of known latency reversal agents in combination with BCL-2 inhibitors (**Table 1**).

**Table 1:** Shock and kill agents and their interactions with BCL-2 and its inhibitors.

Therapeutic agent	Role in shock and kill	Intersection with BCL-2 and its inhibitors	References
PKC Agonists	Latency reversal	BCL-2 Ser70 phosphorylation and activation. Ser70 phosphorylation confers resistance to BCL-2 inhibitor therapy	(9, 57)
Proteasome inhibitors	Latency reversal	Synergistic activity in lymphoma and enhanced anti-HIV activity *in-vitro* with unacceptable ex-vivo toxicity.	(58, 59)
HDAC inhibitors	Latency reversal	Synergistic at high doses and surprisingly antagonistic at lower doses in cutaneous T-Cell lymphoma.	(60)
TLR9 agonists	Latency reversal	TLR-9 signaling causes upregulation of BCL-2 in primary CD4 T-cells. Significantly reduced the cytotoxic potential of BCL-2 inhibition in hairy cell leukemia	(61) (62)
SMAC Mimetics	Latency reversal	Synergistic activity with BCL-2 inhibitors in hepatocellular carcinoma	(63, 64)
IL-15 super agonists	Latency reversal	IL-15 induces altered expression of BCL-2 homologs Bim and MCL-1. N-803 demonstrated no observable anti-HIV effect in an *in-vitro* latency model	(58, 65)
BCL-2 monotherapy	Infected cell cytotoxicity	Selective elimination of HIV infected cells reduced reservoir size Improved action of NK and CD8 cells in human malignancy Improved efficacy of HIV specific CTLs in ex-vivo studies.	(7, 11, 21, 22, 56, 58) (66, 67) (11)
Immune checkpoint blockade	Latency reversal Improved immune function	Improved anti-tumor CD8 activity with combination therapy in cancer	(68)
CAR-T therapy	Infected cell cytotoxicity	Enhanced cytotoxic activity with combination therapy in cancer	(69)

The above table aims to summarize known interactions of drugs being currently investigated for HIV cure, as part of “Shock and Kill” and the BCL-2 protein; and list known effects of combinatorial therapies.

PKC agonists such as bryostatin and prostratin are amongst the most studied latency reversal agents. These drugs have demonstrated latency reversal *in-vitro*, and in *in-vivo* models, though have been disappointing in clinical settings (70, 71). A recent study identified that treatment of CD4 cells with PKC agonists induced phosphorylation on the serine 70 (ser70) residue of BCL-2, that was seen to inhibit the susceptibility of infected cells to cytotoxic stimuli (9). It has been demonstrated that the ser70 phosphorylation can directly inhibit the binding and efficacy of BCL-2 antagonists such as Navitoclax, ABT-737 and Venetoclax (57). These findings suggest that treatment with PKC agonists would likely not allow for maximal clearance of HIV infected cells, necessitating the development of novel PKC agonists which do not have the phosphorylating effect on BCL2. Additionally, the inhibitory effect of the ser70 phosphorylation on BCL-2 inhibitor activity would also likely preclude its clinical efficacy in this specific combination.

Proteasome inhibitors are another class of drugs that have shown latency reversal effect both *in-vitro* and *in-vivo* (22, 58, 72). The combination of the proteasome inhibitor Ixazomib with venetoclax was shown to exhibit strong synergistic activity against lymphoma cells (59). However, in HIV, the combination of proteasome and BCL-2 inhibitors was recently demonstrated to effectively target infected cells in cell line models but was associated with significant toxicity in ex vivo cells (58). A multi-center clinical trial is currently ongoing to determine the safety and efficacy of this combination in patients with multiple myeloma (Clinical trial number NCT03399539), which may inform future efforts at repurposing this combination in setting of HIV.

HDAC inhibitors such as Romidepsin, Vorinostat and Panobinostat have now demonstrated measurable latency reversal in the clinical setting (73–76). In ex vivo studies of cutaneous T cell lymphoma, combination of BCL-2 and HDAC inhibitors was seen to be synergistic at high doses, and paradoxically antagonistic at low doses, suggesting that regulated dosing and maintenance of drug levels may be necessary to achieve good therapeutic efficacy with this combination. Treatment with HDACi also has been shown to lead to the upregulation of the pro-apoptotic BCL-2 family proteins BCL2L11 and BMF (60).

The TLR 9 agonist MGN1703 has demonstrated *in-vivo* latency reversal, while also resulting in immune activation, but had no effect on the latent reservoir (77). TLR9 signaling has previously been demonstrated to result in upregulated expression of BCL-2 in primary activated CD4 cells (61), and it stands to be determined if the absence of reservoir depletion in trials of TLR9 agonists was as a result of BCL-2 upregulation. Consequently, the combination of TLR9 agonism and BCL-2 inhibition may prove an effective combination to target reactivated cells that are apoptosis resistant. However, a recent study involving ex vivo peripheral blood mononuclear cells (PBMCs) from patients with hairy cell leukemia (HCL) revealed that the cytotoxic effects of venetoclax against HCL cells was significantly reduced following TLR9 stimulation with CpG (62).

SMAC mimetics are another group of drugs that have shown efficacy in *in-vivo* animal models (63). The combination of SMAC mimetics and BCL-2 inhibitors have shown synergistic effect in hepatocellular carcinomas (64). This combination therefore may represent a possible combination to target the latent reservoir.

The IL-15 super-agonist *N-803* was demonstrated to cause latency reversal and reservoir depletion in SIV infected macaques (78), and has recently demonstrated safety in a phase I clinical trial (79). IL-15 has been noted to regulate T-cell survival by altering the expression of Bim and MCL-1, overall favoring a pro-survival phenotype (65). In line with this observation, the combination of IL-15 and Venetoclax did not result in any observable decline in the number of HIV infected cells in an *in-vitro* model of latency (58). Further studies are required to assess if this combination would prove efficacious *in vivo*.

## The “kill”: BCL-2 inhibitors and immune effector potentiation

Immune function is a critical determinant of HIV reservoir size but varies widely, with the majority of infected individuals succumbing to a combination of viral escape mutations and host immune exhaustion, ultimately allowing for the persistence of HIV infection (80–82). The second pillar of the “shock and kill” strategy centers around the clearance of infected cells by immune effector cells and anti-retroviral therapy.

As monotherapy BCL-2 inhibition has been shown to independently result in the selective elimination of HIV infected cells and decrease the size of the HIV reservoir in *in-vitro* and ex-vivo studies (7, 11, 21, 22, 56, 58). BCL-2 inhibition has been shown to improve the efficacy of both NK cells and CTLs in human malignancies (66, 67). BCL-2 inhibition has now demonstrated measurably improved clearance of infected cells by CTLs in *in-vitro* and ex-vivo models of HIV (11), though further studies are required to demonstrate similar results in *in-vivo* settings.

Immune checkpoint blockade in HIV has demonstrated improved HIV specific CD8 degranulation *in vitro* (83, 84). Modest improvement in CD8 function has also been demonstrated *in vivoin vitro* (85). In human malignancy, the combination of immune checkpoint blockade and BCL-2 inhibition was seen to improve anti-tumor CD8 activity (68), suggesting that this combinatorial approach may also help improve anti-HIV immune function, though no studies have examined this combination to date.

HIV specific chimeric antigen receptor (CAR) T cells have demonstrated promise as a viable treatment option for HIV, having demonstrated efficacy at clearing HIV *in-vitro* and *in-vivo*, demonstrating the ability to traffic to lymphoid tissues and reduce the viral reservoir (86–89). BCL-2 inhibition has been demonstrated to improve the efficacy of CAR-Ts in the setting of malignancy (69), suggesting that combinations in HIV may allow for superior clearance, however further investigations are required to study this combination.

## Conclusion

Successful Shock and kill therapy for HIV will ultimately require a potent and effective combination of latency reversal and immune function. Evidence thus far suggests that BCL-2 is a critical determinant of HIV survival and persistence. BCL-2 inhibition allows for the selective elimination of HIV infected cells, both as a monotherapy, and in combination with host immune cells, suggesting that targeted BCL-2 inhibition may lead to the potentiation of shock and kill treatment regimens, and ultimately HIV cure.

## Author contributions

APC organized and wrote the manuscript. APC and ADB were involved in conceptualization, review and final drafting. Both authors contributed to the article and approved the submitted version.

## Funding

Portions of this work were funded through grants (grants AI110173 and AI120698) from the National Institute of Allergy and Infectious Diseases of the NIH and the Mayo Clinic Foundation. The opinions expressed are solely of the authors and do not necessarily represent the opinions of the funding organization(s).

## Acknowledgments

We thank the innumerable persons who have participated in research studies, without which many of the described observations would not have been possible. **Figure 1** was created using Biorender.com

## Conflict of interest

ADB is supported by grants from NIAID grants AI110173 and AI120698 Amfar #109593 and Mayo Clinic HH Sheikh Khalifa Bin Zayed Al-Nahyan Named Professorship of Infectious Diseases. ADB is a paid consultant for Abbvie, Gilead, Freedom Tunnel, Pinetree therapeutics Primmune, Immunome, MarPam, Rion, Symbiosis, NexImmune and Flambeau Diagnostics, is a paid member of the DSMB for Corvus Pharmaceuticals, Equilium, CSL Behring, and Excision Biotherapeutics, has received fees for speaking for Reach MD, Peer Voice, and Medscape, owns equity for scientific advisory work in Tier 1 Bio, Zentalis, Rion, and Nference, and is founder and President of Splissen therapeutics, and Member of the Board of Attivare.

The remaining author declares that the research was conducted in the absence of any commercial or financial relationships that could be construed as a potential conflict of interest.

## Publisher’s note

All claims expressed in this article are solely those of the authors and do not necessarily represent those of their affiliated organizations, or those of the publisher, the editors and the reviewers. Any product that may be evaluated in this article, or claim that may be made by its manufacturer, is not guaranteed or endorsed by the publisher.
